# X-ray Phase Contrast Imaging of Cell Isolation with Super-Paramagnetic Microbeads

**DOI:** 10.1371/journal.pone.0045597

**Published:** 2012-09-24

**Authors:** Rongbiao Tang, Wei-Min Chai, Guo-Yuan Yang, Honglan Xie, Ke-Min Chen

**Affiliations:** 1 Department of Radiology, Rui Jin Hospital, Shanghai Jiao Tong University School of Medicine, Shanghai, People’s Republic of China; 2 Neuroscience and Neuroengineering Center, Med-X Research Institute, Shanghai Jiao Tong University, Shanghai, People’s Republic of China; 3 Shanghai Institute of Applied Physics, Chinese Academy of Sciences, Shanghai, People’s Republic of China; Northwestern University Feinberg School of Medicine, United States of America

## Abstract

Super-paramagnetic microbeads are widely used for cell isolation. Evaluation of the binding affinity of microbeads to cells using optical microscopy has been limited by its small scope. Here, magnetic property of microbeads was first investigated by using synchrotron radiation (SR) in-line x-ray phase contrast imaging (PCI). The cell line mouse LLC (Lewis lung carcinoma) was selected for cell adhesion studies. Targeted microbeads were prepared by attaching anti-VEGFR2 (vascular endothelial growth factor receptor-2) antibody to the shell of the microbeads. The bound microbeads were found to better adhere to LLC cells than unbound ones. PCI dynamically and clearly showed the magnetization and demagnetization of microbeads in PE-50 tube. The cells incubated with different types of microbeads were imaged by PCI, which provided clear and real-time visualization of the cell isolation. Therefore, PCI might be considered as a novel and efficient tool for further cell isolation studies.

## Introduction

Cell isolation is an important technique for therapy or research. Microbeads are designed for cell isolation with high binding capacity and good magnetic property [1].The specific antibodies conjugated onto the surface of the magnetic microbeads can be used to specifically recognize their target proteins expressed in cells [2], [3]. Clinically, if metastatic cancer cells are diagnosed accurately at an early stage, they may be treated effectively. The bound microbeads with specific marker can help detect circulating tumor cells (CTCs) in the blood or body fluids. The high-affinity binding of microbeads enhances the sensitivity of cancer cell detection. Accordingly, it is very important to estimate the binding affinity of the bound microbeads to their target before their clinical application. Optical microscopy is often used to figure out the magnetic beads and clarify their capability in cell isolation; however, the imaging method could only be used for observing the surface of the specimens. In addition, the field of view for optical microscopy is relatively narrow.

**Figure 1:**
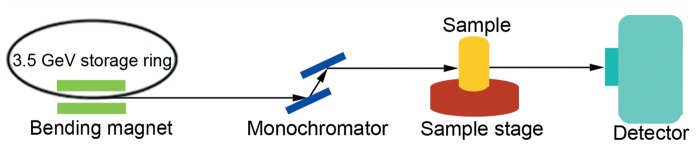
The experimental setup for in-line PCI at BL13W1 in SSRF.

To overcome the disadvantages of light microscopy, novel imaging method should be introduced. Synchrotron radiation (SR) is characterized by its high brightness, high intensity and highly collimated [4]. SR imaging (SRI) offers high spatial resolution down to the sub-micron scale. Also, SRI could provide millisecond-level temporal resolution; as a result, it captures clear images of rapidly moving objects. Besides absorption, phase shift is another important contrast mechanism between x-rays and tissues. Phase contrast imaging (PCI), utilizing the phase shift, is approximately 1000 times more sensitive than conventional absorption imaging [5]. Therefore, phase contrast imaging is often used to enhance the contrast, especially when the absorption is weak [6], [7], [8].

It is widely accepted that biomolecules are essential for tumor growth, invasion and metastasis [9], [10], [11]. Among them, VEGFR2 (vascular endothelial growth factor receptor-2) is an important positive regulator of cell migration and angiogenesis [12]. VEGF (vascular endothelial growth factor) binding to VEGFR2 activates downstream signaling transduction pathways, resulting in cell proliferation and migration [13], [14]. VEGFR2 is highly expressed on tumor endothelial cells and has been detected in many cancer cell lines [12], [13]. Therefore, VEGFR2 may play an important role in cell identification and isolation.

**Figure 2:**
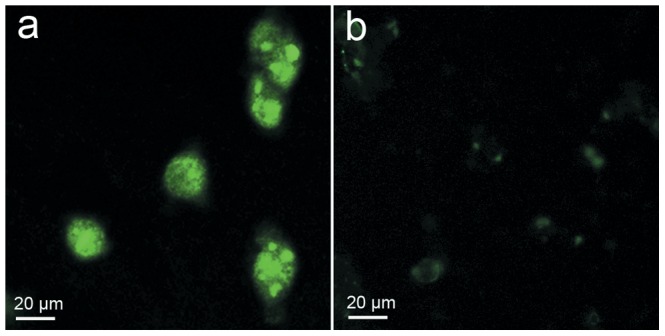
Fluorescent microscopy images of LLC cells incubated with primary and fluorescent secondary antibodies (a), or with secondary antibody only (b). Note slight non-specific fluorescence in b.

In this study, we fabricated anti-VEGFR2 antibody-loaded microbeads, and first used PCI to noninvasively image cell isolation with microbeads. Additionally, the magnetization and demagnetization of microbeads were also dynamically investigated.

## Materials and Methods

### Cell Line

Mouse Lewis lung carcinoma (LLC) cells were purchased from Chinese Academy of Sciences in Shanghai. LLC cells were cultured in Dulbecco’s modified Eagle’s medium (DMEM) supplemented with 10% fetal bovine serum (FBS), penicillin (100 U/ml), and streptomycin (100 µg/ml). Cells were incubated at 37°C in a humidified atmosphere containing 5% CO_2_.

**Figure 3:**
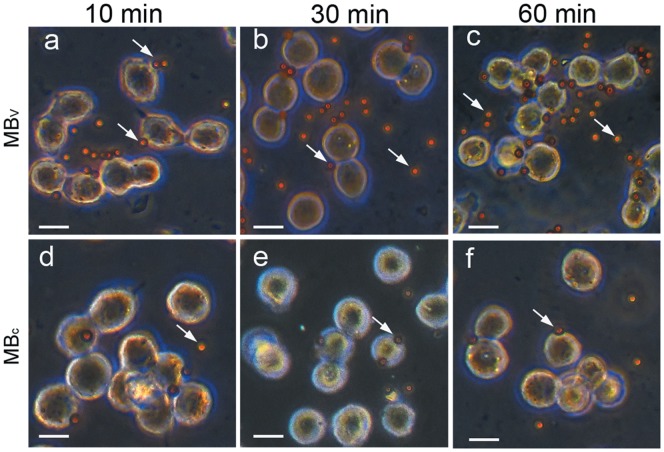
Representative optical microscopic images of LLC cells following incubation with MB_V_ (a–c) or MB_c_ (d–f) for 10 (a and d), 30 (b and e), 60 min (c and f). MB_V_ preferentially attached to LLC cells, MB_c_ rarely attached to the cells. Arrows indicate microbeads; original magnification, ×400; bars: 20 µm.

### Anti-VEGFR2 Conjugated Microbeads Preparation

VEGFR2-targeted microbeads (MB_V_) were obtained according to manufacturer’s instructions. Briefly, 0.3 ml superparamagnetic microbeads (4.5 µm, Dynabeads M-450, Invitrogen) were transferred to a tube. The tube was placed in a magnet for 1 min, and then the supernatant was discarded. After washed once in 0.1 M sodium phosphate buffer (pH 7.4), the microbeads were resuspended in 0.2 ml rat anti-mouse VEGFR2 monoclonal antibody (eBioscience), and incubated at 37°C on an orbital shaker for 24 hours. Finally, the targeted microbeads were washed thrice with phosphate buffered saline (PBS) containing 0.1% (w/v) bovine serum albumin (BSA) and 2 mM EDTA, and then stored at 4°C.

**Figure 4:**
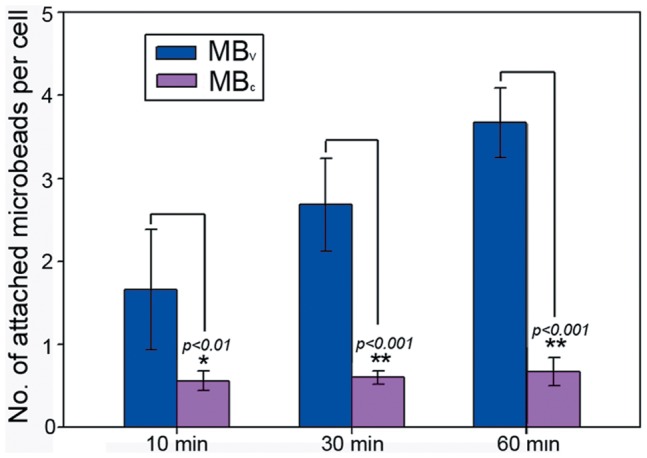
Attachment of MB_V_ or MB_c_ to LLC cells in cell culture experiments. The binding of MB_V_ to LLC cells was more efficient than that of MB_c_. The binding affinity of MB_V_ increased over a 60 minute time period. Error bars  =  standard deviations.

**Figure 5:**
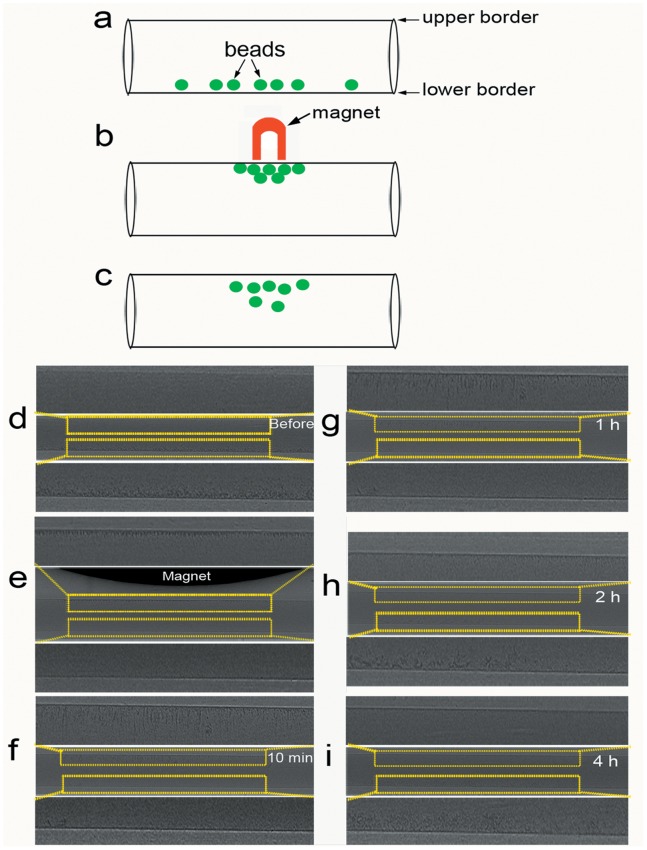
PCI of super-paramagnetic microbeads under the influence of a magnetic field. (a–c) Schematic drawings of magnetization and demagnetization of microbeads. (d) Before introducing the magnetic field, the microbeads were clearly visualized along the lower border of the PE-50 tube. (e) The microbeads assembled along the upper edge of the PE-50 tube by placing a permanent magnet over the tube. (f–i) The magnetism of the microbeads attenuated markedly after removing the magnet, and then the beads gradually fell from the upper border over the following 4 hours. Images were obtained at the energy of 14 keV. The pixel size was 0.74 µm ×0.74 µm.

### Cells Immunostaining

LLC cells (1×10^5^) were grown on different glass coverslips and cultured for 24 h. Cells were fixed in 4% paraformaldehyde (w/v in PBS) for 20 min, washed in PBS and blocked in 10% FBS, and then incubated with a rabbit anti-mouse VEGFR2 antibody (1∶300; Santa Cruz Biotechnology, sc-504) for 1 h at 37°C. Then the cells were washed in PBS and incubated with Alexa-Fluor 488-labeled goat anti-rabbit secondary antibody (Invitrogen, Switzerland) for 1 h. Coverslips were washed with PBS twice, mounted on glass microscope slides and then viewed under a Leica DM2500 microscope. Cells stained with secondary antibody only were used as controls.

**Figure 6:**
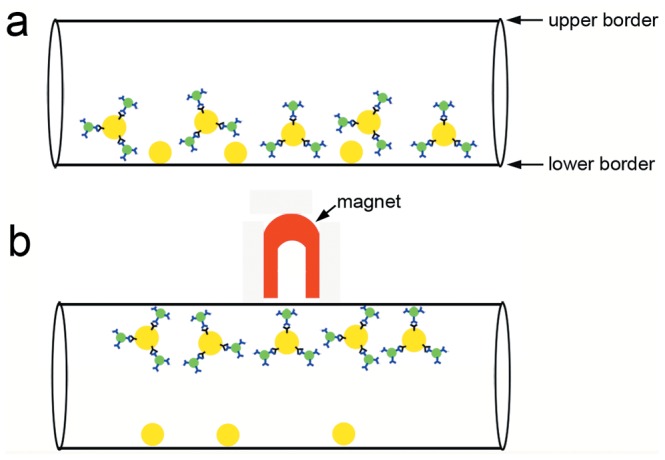
A cartoon depiction of MB_V_ binding to LLC cells. The anti-VEGFR-2 antibody (blue)-conjugated microbeads (green) specially bound to VEGFR2 protein (black) expressed on the surface of LLC cells (yellow). Then the bead-bound LLC cells are attracted to the magnet and separated from unbound cells.

### Cell Adhesion Studies

LLC cells (1×10^5^) were grown on glass coverslips in six-well plates for 48 h. Nonlabeled control microbeads (MB_C_) were washed thrice with PBS containing 0.1% (w/v) BSA and 2 mM EDTA before they were used. MB_C_ or MB_V_ were suspended at a concentration of 10^8^/ml in DMEM. Then LLC cells were incubated with 8×10^6^ MB_C_ or 8×10^6^ MB_V_ at 37°C for 10, 30 or 60 min. Cells were washed five times with DMEM to remove unbound microbeads. The number of the beads adhered to the cell surface was counted using the 40×optical lens in five randomly chosen fields.

**Figure 7:**
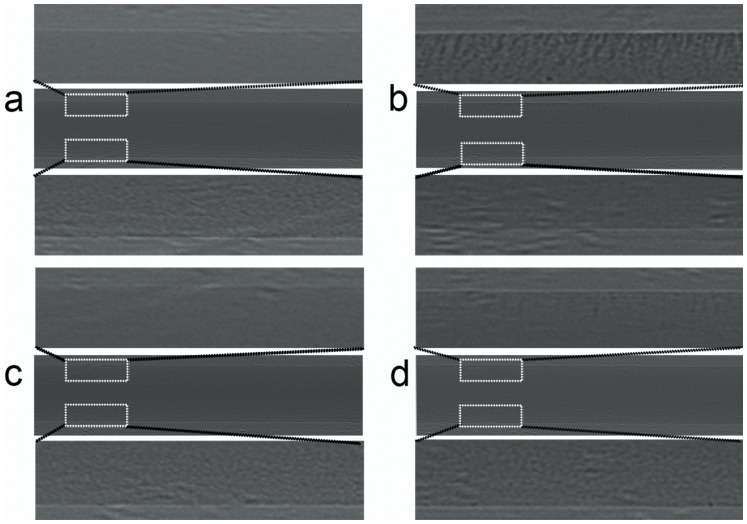
PCI of LLC cells isolation with MB_V_ (a–b) or MB_C_ (c–d). (a and c) Before introducing the magnetic field, the mixture of cells and microbeads were clearly revealed along the lower edge of the PE-50 tube. (b and d) After placing a magnet over the tube, the bead-bound cells were attracted towards the upper edge of the tube. Note that the MB_V_-bound cells (b) were much more than MB_C_-bound ones (d). Images were obtained at the energy of 14 keV. The pixel size was 0.74 µm ×0.74 µm.

### SR Parameters

Imaging was performed at the beamline BL13W1 of the Shanghai Synchrotron Radiation Facility (SSRF) in China. X-rays were derived from an electron storage ring with an accelerated energy of 3.5 GeV, and an average beam current of 180 mA. X-rays were monochromatized by a double-crystal monochromator with Si (111) and Si (311) crystals. The energy resolution was ΔE/E <5×10^−3^. Images were obtained by using a (100 µm thick) CdWO_4_ cleaved single crystal scintillator and a CCD camera (Photonic Science, UK). Samples was placed 34 m downstream of the synchrotron source, and the distance between the sample and the CCD camera had a changeable range of 8 m (**Fig. 1**).

### Image Acquisition

25 µl original microbeads solution was diluted with PBS to form a 10^7^/ml solution. About 20 µl dilute solution (2×10^5^ microbeads) was placed in a PE-50 tube. Phase contrast images of microbeads were obtained using a CCD camera with the resolution of 0.74 µm. Imaging was performed at the energy level of 14 keV, and with the object-detector distance of 30 cm. In order to image the magnetism of the microbeads, a magnet was placed over the PE-50 tube. LLC cells were washed five times with PBS, treated with trypsin and then transferred to a PE-50 tube after incubated with MB_V_ or MB_C_ for 1 h. Cell isolation was also imaged by placing a magnet over the PE-50 tube.

### Statistical Analysis

Data were reported as means ± standard deviations. Statistical analysis was performed using Student’s t-test. A *p* value of <0.05 was considered statistically significant.

## Results

### Cell Culture Experiments

Immunocytochemical staining for VEGFR2 expression was positive for LLC cells (**Fig. 2**). The MB_V_ were found to better bind to LLC cells compared with MB_C_ (**Fig. 3**). In **Fig. 4**, numbers of attached MB_V_ per cell after 10, 30, 60 min incubation were 1.66±0.72, 2.68±0.56 and 3.67±0.42, respectively. The corresponding values for MB_C_ were 0.56±0.12, 0.60±0.08 and 0.67±0.17, respectively. Adhesion of MB_V_ to LLC cells was significantly higher than MB_C_ at each time point. The binding affinity of MB_V_ increased over a 60 minute time period.

### PCI of Microbeads

Three cartoons depict the attraction of super-paramagnetic microbeads to a magnet (**Fig. 5a–c**). On phase contrast image, the microbeads were clearly visualized along the lower border of the PE-50 tube (**Fig. 5d** **)**. After a magnet was placed over the tube, the microbeads were attracted towards the magnet (**Fig. 5e** **)**, which demonstrates that these microbeads display magnetic properties when placed in a magnetic field. With the removal of the magnet, the magnetism of the microbeads attenuated markedly, and then the beads gradually fell from the upper border over the following 4 hours (**Fig. 5f–i**).

### PCI of LLC Cells Isolation

The principles of cell isolation studies are described in **Fig. 6**. The bead-bound LLC cells are attracted to the magnet and separated from unbound cells. As shown in **Fig. 7**, PCI could provide clear visualization of the cell isolation procedure. **Fig. 7a** and **Fig. 7c** are phase contrast images of LLC cells incubated with MB_V_ or MB_C_, respectively. The cells and beads sank and assembled along the lower edge of the PE-50 tube. When exposed to a magnetic field, the bead-bound cells clustered together through magnetic attraction (**Fig. 7b and 7d**). Phase contrast images clearly showed that the number of MB_V_-bound cells (**Fig. 7b**) was much higher than that of MB_C_-bound ones (**Fig. 7d**).

## Discussion

The aim of this study was to assess the feasibility of using PCI to investigate the magnetic property and affinity of microbeads. Our results have suggested that PCI is a feasible, non-invasive, and real-time tool for imaging cell isolation.

Paramagnetic microbeads can serve as good delivery carriers for biomolecules [15]. Desired antibodies could be incorporated onto the surface of microbeads. Then the antibody-loaded microbeads specially bind to corresponding proteins from cells or tissues. VEGFR2 has been shown to play a crucial role in the development and progression of cancer [12]. Imaging VEGFR2 could provide insights into the further evaluation of tumor angiogenesis [16], [17]. Here, anti-VEGFR2 antibody was bound to the surface of microbeads. Attachment of different types of microbeads was assessed in cell culture experiments using LLC cells that express VEGFR2, as confirmed by immunocytochemical analysis. The anti-VEGFR2 antibody-conjugated microbeads could specially bind to VEGFR2 protein expressed on the surface of LLC cells. The binding affinity of MB_V_ to VEGFR2, which increased over a 60 minute time period, was much higher than that of MB_C_. Therefore, MB_V_ may become a potential probe for identification of VEGFR2. Additionally, we have provided the first evidence suggesting that MB_V_ preferentially bind to LLC cells when compared with MB_C_.

PCI, utilizing the phase shift, can provide high sensitivity to weakly absorbing materials [5], [16], [18]. In **Fig. 5**, magnetization and demagnetization of microbeads could be clearly displayed in real-time using PCI. Because the gravity of microbeads was high compared with PBS, they sank and assembled along the lower edge of the tube (**Fig. 5d**). Magnetic property is an important characteristic for microbeads when they are placed in an external magnetic field. Microbeads became magnetized when they were exposed to a permanent magnet (**Fig. 5e**). The magnetic attraction for microbeads was greater than their gravitational attraction, so they rose and clustered together along the upper edge of the PE-50 tube. As the magnet was withdrawn far away, the induced magnetism of the microbeads became weaker and weaker until finally they were completely demagnetized. Due to their gravitational attraction, the microbeads began to fall from the upper border. However, the sedimentation rate of the magnetized microbeads appeared to be much smaller than unhandled microbeads. This may be because the microbeads retained little residual magnetism when removed from the magnetic field.

Microbeads provide a versatile tool for bio-magnetic separations. They are also often added to the sample as a tracer to evaluate the success of injections [19]. Cells can be analyzed and isolated on the basis of antibody-antigen binding. The specific binding increases the efficiency and accuracy of cell isolation [2], [3]. In the cell culture experiments, adherence of MB_V_ to LLC cells was 8.8 times higher compared to MB_C_ after 1 h incubation. The LLC cells bound with magnetized MB_V_ will be attracted to the magnet, and thus they could be separated from the unbound cells. Along the upper edge of the tube, the beads-bound cells were clearly shown by PCI (**Fig. 7b**). Because most of the LLC cells were bound with MB_V_, few cells retained along the lower edge of the tube. However, only a very few MB_C_-bound cells could be found along the upper edge of the tube, and most cells retained along the lower edge of the tube (**Fig. 7d**).

To conclude, our study is the first to dynamically and clearly display the magnetization and demagnetization procedure of microbeads by using PCI. The VEGFR2 antibodies obviously increase the adhesion of microbeads to LLC cells. The physical (good magnetism) and biological (high binding capacity) characteristics of microbeads contribute to their superior performance in cell isolation. PCI technique facilitates the non-invasive imaging of the cell isolation, which provides a good way of estimating the binding affinity of the bound microbeads to their target. Therefore, PCI may become a novel, promising imaging tool for future studies of *in vitro* cell culture or *in vivo* experiments.
